# Cardiopulmonary Exercise Testing-Guided Exercise Therapy in Hypertensive Patients: A Single Center Study

**DOI:** 10.1155/2024/8476971

**Published:** 2024-08-08

**Authors:** Qin Lu, Jingjing Lu, Che Li, Ping Huang, Fenfen Jiang, Xia Zhao, Jianqin Zhang, Yi Huang, Zhenliang Chu

**Affiliations:** ^1^ Department of Cardiology The Second Affiliated Hospital of Jiaxing University, Jiaxing, Zhejiang, China; ^2^ Health Management Center The Second Affiliated Hospital of Jiaxing University, Jiaxing, Zhejiang, China

**Keywords:** blood pressure variability, cardiopulmonary exercise testing, cardiorespiratory reserve, hypertension

## Abstract

**Objective:** To observe the effects of cardiac rehabilitation guided by cardiopulmonary exercise testing (CPET) on cardiorespiratory reserve function, blood pressure, blood pressure variability, and lipid metabolism in patients with hypertension.

**Methods:** A randomized trial enrolled 67 Grade 1 hypertensive patients on antihypertensive drugs, divided into conventional (*n* = 35) and CPET (*n* = 32) groups. Antihypertensive drugs were not adjusted in both groups during the study period. Blood pressure, cardiorespiratory indicators, lipid profile, and BMI were assessed pre/post 12 weeks.

**Results:** Postintervention, the CPET group exhibited significantly lower blood pressure levels and improved cardiac indicators compared to the conventional group (*p* < 0.05). CPET group showed greater improvements in cardiorespiratory endurance indicators (*p* < 0.05). The cardiorespiratory endurance indicators showed significantly greater increases in the CPET group compared to the conventional group (*p* < 0.05). Low-density lipoprotein cholesterol (LDL-C), total cholesterol (TC), triglycerides (TG), and body mass index (BMI) were significantly lower in the CPET group (*p* < 0.05).

**Conclusion:** In addition to drug treatment, cardiac rehabilitation guided by CPET can effectively improve blood pressure control, reduce blood pressure variability, improve cardiorespiratory function and lipid metabolism, and increase exercise endurance in patients with Grade 1 hypertension. Its efficacy is clear and safe, with clinical value for promotion.

## 1. Introduction

Hypertension is a common chronic cardiovascular disease in clinical practice and is one of the important risk factors for inducing cardiovascular and cerebrovascular diseases. Poorly controlled hypertension is a significant factor in serious cardiovascular events and even death [1–3]. Therefore, improving the control rate of hypertension is crucial. Large-scale meta-analyses have shown that exercise training can reduce systolic blood pressure (SBP) by 6−8 mmHg (1 mmHg = 0.133 kPa) and diastolic blood pressure (DBP) by 3−5 mmHg in hypertensive patients [4, 5]. The cardiopulmonary exercise test (CPET) is an important examination method for the clinical assessment of cardiac rehabilitation in hypertensive patients. It is a noninvasive test method for objectively evaluating cardiorespiratory reserve function and exercise endurance. CPET can accurately determine indicators such as anaerobic threshold and maximum oxygen uptake in patients, distinguish the reasons for exercise limitation in different disease patients, and provide personalized exercise prescriptions [6]. In this study, based on the assessment of the overall functional status of elderly individuals, individualized moderate exercise rehabilitation prescriptions were formulated for the treatment of hypertension according to CPET results. The effects of exercise on lowering dynamic blood pressure, controlling blood lipids, and improving cardiopulmonary function in elderly hypertensive patients were observed, achieving good results.

## 2. Objectives and Methods

### 2.1. Study Population

**Figure 1:**
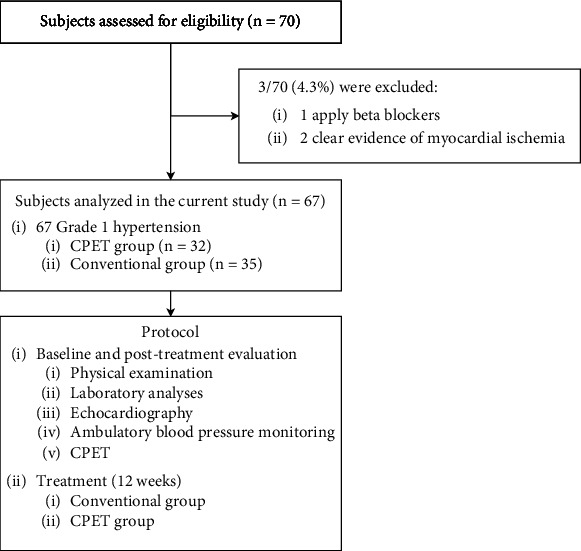
Enrolment flow diagram and the protocol. CPET, cardiopulmonary exercise testing.

A total of 70 patients diagnosed with Grade 1 hypertension in the Cardiology Department of our hospital from January 2023 to June 2023 were prospectively recruited. They were divided into the CPET group and the conventional group, with 35 patients in each group. Three patients dropped out from the CPET group.  Figure 1 shows the enrolment flow diagram and the protocol. There were no statistically significant differences in gender, age, duration of illness, hemoglobin levels, or types of medication between the two groups (*p* > 0.05), as shown in Table 1. Inclusion criteria: (1) diagnosed according to the “Chinese Guidelines for the Prevention and Treatment of Hypertension (2018 Revised Edition)” [7]; (2) blood pressure controlled with antihypertensive medication to within 140/90 mmHg; (3) age ≥60 years old; (4) no regular exercise or physical activity in the past 6 months; (5) no adjustment of antihypertensive medication regimen in the past month; (6) able to adhere to exercise regimen; (7) informed consent signed by participants who were aware of and agreed to participate in the study. Exclusion criteria: (1) secondary hypertension; (2) hypertensive emergencies, severe hypertension, or hypertensive crises; (3) coronary heart disease or clear evidence of myocardial ischemia during exercise testing; (4) various types of cardiomyopathy and valvular heart disease; (5) clinical symptoms of heart failure, signs, and/or left ventricular ejection fraction (LVEF) <50% on echocardiography; (6) left ventricular mass index (LVMI) ≥110 g/m^2^; (7) pulmonary function abnormalities due to pulmonary diseases; (8) anemia, thyroid dysfunction, stroke, severe hepatic or renal dysfunction, or other acute illnesses; (9) inability or unsuitability for exercise due to other reasons; (10) use of beta-blockers; (11) resistant hypertension. This study was approved by the Medical Ethics Committee of our institution, and informed consent was obtained from all patients.

### 2.2. Methods

#### 2.2.1. Medical History Collection and Physical Examination

All patients underwent measurements of clinic blood pressure, heart rate, height, weight, and body mass index (BMI), and their medical history, including the duration of hypertension and presence of complications such as hyperlipidemia, was recorded.

#### 2.2.2. Laboratory Examinations

Fasting peripheral venous blood samples were collected from patients before and after the intervention to measure serum levels of blood lipids, including low-density lipoprotein cholesterol (LDL-C), total cholesterol (TC), and triglycerides (TG).

#### 2.2.3. Echocardiography

Echocardiography was performed using a color Doppler ultrasound diagnostic instrument (Vivid E95, GE Healthcare) to assess LVEF, left ventricular interventricular septum (LVST), left ventricular posterior wall (LVPWT), left ventricular end-diastolic dimension (LVEDD), LVMI, early maximal ventricular filling velocity/atrial maximal ventricular filling velocity (E/A ratio), and left atrial diameter (LAD).

#### 2.2.4. Ambulatory Blood Pressure Monitoring

Both the conventional group and the CPET group were observed and analyzed for dynamic blood pressure parameters with blood pressure monitoring (ABPM-021, China) before and after the 12-week intervention, and comparisons were made between and within groups. Diagnostic criteria for hypertension based on dynamic blood pressure monitoring refer to the “Chinese Guidelines for the Prevention and Treatment of Hypertension (2018 Revised Edition)” [7]: 24 h average blood pressure ≥ 130/80 mmHg, daytime average blood pressure ≥ 135/85 mmHg, and nighttime average blood pressure ≥ 120/70 mmHg. Dynamic blood pressure parameters included resting SBP (rSBP), resting DBP (rDBP), 24 h SBP (24hSBP), 24 h DBP (24hDBP), daytime SBP (dSBP), daytime DBP (dDBP), nighttime SBP (nSBP), and nighttime DBP (nDBP).

#### 2.2.5. CPET

A CPET system (utilizing the German CareFusion exercise testing system, with gas provided by Shanghai Baolai Gas Co., Ltd.) was used for the evaluation of 12-lead electrocardiography, oxygen saturation, noninvasive blood pressure, and gas exchange function. Specifically, patients first underwent seated static pulmonary function tests, followed by symptom-limited bicycle exercise on a cycle ergometer while monitoring 12-lead electrocardiography, oxygen saturation, noninvasive blood pressure, and various cardiorespiratory reserves. The modified BRUCE protocol was used for symptom-limited CPET with variable speed and incline [8]. Exercise endpoints included: (1) achieving the target heart rate; (2) development of typical angina or positive ST-segment ischemic changes; (3) exercise-induced SBP > 230 mmHg or DBP > 115 mmHg, or a decrease in blood pressure > 10 mmHg, or occurrence of severe arrhythmias; (4) severe peripheral circulatory insufficiency during exercise, such as cyanosis, wheezing, pallor, nausea, or neurological symptoms such as ataxia or dizziness; (5) subjective exhaustion preventing maintenance of the set cycling speed [9].

Changes in CPET parameters were observed and analyzed in both the conventional group and the CPET group before and after the 12-week intervention, during which cardiorespiratory reserve function indicators and exercise endurance indicators were monitored and recorded. Exercise endurance indicators included forced expiratory volume in 1 s/forced vital capacity (FEV1/FVC%), peak heart rate (HR peak), peak SBP (SBP peak), peak DBP (DBP peak), anaerobic threshold oxygen uptake (AT-vo2), maximum oxygen uptake (Max-vo2), maximum oxygen uptake per kilogram (Max-vo2/Kg), anaerobic threshold oxygen uptake per kilogram (AT-vo2/Kg), maximum metabolic equivalent (Max-Mets), peak metabolic equivalent (Mets peak), anaerobic threshold metabolic equivalent (AT-Mets), maximum oxygen pulse (Max-vo2/HR), anaerobic threshold oxygen pulse (AT-vo2/HR), peak oxygen consumption (VO2 peak), and maximum power (power peak).

During the test, patients rested quietly on the cycle ergometer for 3 min, followed by a 3-min unloaded warm-up at 60 revolutions per minute (rpm). Subsequently, the workload was increased at a rate of 20–30 W per minute, adjusted based on patient age and sex, aiming for symptom-limited maximal effort within 6–10 min to determine maximal exercise capacity. After exercise rehabilitation, patients remained seated quietly for a recovery period of 3–5 min [10]. Individualized exercise prescriptions were formulated based on exercise testing guidelines. [11]

### 2.3. Treatment Plan

#### 2.3.1. Conventional Group

Patients in the conventional group maintained their usual antihypertensive medication regimen throughout the study period without any adjustments. They received health education and dietary guidance aimed at controlling salt and fat intake and were advised to engage in moderate exercise.

#### 2.3.2. CPET Group

Patients in the CPET group received individualized exercise interventions based on objective quantitative assessment from CPET. Treatment plan: Walking exercise was selected for the CPET group. The specific implementation of exercise intensity, duration, frequency, and period was as follows: (1) calculation of individualized exercise intensity. The exercise intensity was moderate, with each patient's intensity calculated based on their Max-vo2 from CPET results. The target heart rate was set at 40%–60% of Max-vo2, with the Borg scale ratings of 11–13 indicating a perceived fatigue level from light to somewhat heavy during exercise. (2) Exercise duration, frequency, and period: Patients adhered to 30 min of walking exercise daily, with a 5-min warm-up before and 5-min recovery after exercise, totaling 40 min. Exercise frequency was 5 days per week, and the exercise period was 12 weeks.

#### 2.3.3. Quality Control

Data collection was performed by the researchers reviewing patient records, and CPET was conducted under the guidance of cardiac rehabilitation physicians. Exercise prescriptions were adjusted based on target heart rates, and case managers followed up on exercise rehabilitation training execution. Laboratory parameters, echocardiography, CPET assessments, and patient readiness for exercise were reassessed before and after 12 weeks of intervention. Patients were provided with exercise wristbands to monitor their safe heart rates during exercise. To maintain effective cardiac rehabilitation outcomes, the use of the *Cardiac Rehabilitation Exercise Self-Management Manual* was employed to record exercise modalities, duration, heart rates, and adverse reactions. All echocardiography examinations were conducted by specialized cardiac ultrasound physicians, and individualized exercise prescriptions were jointly formulated and implemented by a cardiac rehabilitation subspecialist attending physician and a cardiovascular specialist chief physician.

### 2.4. Statistical Methods

Statistical analysis was conducted using SPSS 26.0 software. Continuous data were expressed as mean ± standard deviation (SD). Paired *t*-tests were used for within-group comparisons, and independent sample *t*-tests were used for between-group comparisons. Categorical data were expressed as percentages and analyzed using Pearson's chi-square test. Fisher's exact chi-square test was used for frequencies ≤ 1. Nonparametric rank sum tests were used for ordinal data. A significance level of *p* < 0.05 was considered statistically significant.

## 3. Results

### 3.1. Comparison of 24-h Dynamic Blood Pressure Before and After Intervention in CPET Group and Conventional Group

Before the intervention, there was no statistically significant difference in blood pressure between the CPET group and the conventional group (*p* > 0.05). Both the CPET group and the conventional group showed a decrease in blood pressure before and after intervention, with statistically significant differences observed within each group (*p* < 0.01). After the intervention, there was a statistically significant difference between the two groups (both *p* < 0.05). Refer to Table 2 for details.

### 3.2. Comparison of Echocardiographic Parameters Before and After Intervention in Both Groups

Compared to before intervention, all parameters significantly improved in the CPET group after intervention and were superior to those in the conventional group after intervention (all *p* < 0.05). See Table 3.

### 3.3. Comparison of CPET Parameters Before and After Intervention in Both Groups

Compared to before intervention, all CPET parameters significantly changed in the CPET group after intervention and were higher than those in the conventional group after intervention (all *p* < 0.05). See Table 4.

### 3.4. Comparison of Lipid Metabolism and BMI Before and After Intervention in Both Groups

Compared to before intervention, the levels of various indicators significantly decreased in the CPET group after intervention and were lower than those in the conventional group after intervention (all *p* < 0.05). See Table 5.

## 4. Discussion

CPET is an indispensable assessment technique throughout cardiac rehabilitation programs. Exercise intervention is a crucial aspect of hypertension management, often regarded as one of the key pillars in hypertension interventions [12]. With the advancement of modern medicine, healthcare professionals are increasingly emphasizing personalized and quantified exercise recommendations for hypertensive patients. Exercise training can attenuate sympathetic nervous system activity, increase capillary density or quantity, reduce blood viscosity, and optimize vascular wall relaxation function, thus lowering blood pressure [13, 14]. The 2020 European Society of Cardiology (ESC) guidelines on sports cardiology and cardiovascular disease patients once again underscore the importance of exercise in controlling blood pressure in hypertensive patients, elucidating the antihypertensive effects of various types of exercise [15]. However, evidence regarding whether exercise can also reduce dynamic blood pressure is limited. In this study, before intervention, there was no statistically significant difference in blood pressure between the CPET group and the conventional group (*p* > 0.05). Both groups showed a decrease in blood pressure before and after intervention, with statistically significant differences observed within each group (*p* < 0.01), and there were statistically significant differences between the two groups after intervention (both *p* < 0.05). Patients with Grade 1 hypertension showed a significant reduction in blood pressure indicators after 12 weeks of home-based rehabilitation exercise in the CPET group compared to before intervention, which is consistent with the results of Igarashi and Nogami [16]. Furthermore, Pescatello et al. [17] highlighted through meta-analysis that after 10 weeks of regular exercise, systolic and DBP decreased by 13 and 18 mmHg, respectively, in elderly hypertensive patients, which is largely in line with the findings of this study. Additionally, patient adherence to exercise, regular aerobic exercise based on target heart rate, and periodic follow-up with adjustments to exercise prescriptions have been shown to improve the condition of hypertension.

This study demonstrates that, compared to preintervention levels, the echocardiographic indicators of both groups showed significant improvement after intervention, with the CPET group exhibiting superior results to the conventional group postintervention (all *p* < 0.05). These findings are consistent with the study by Noone et al. [18], suggesting that cardiac rehabilitation intervention can significantly improve cardiovascular and skeletal muscle function in hypertensive patients [19]. CPET assessment revealed that, compared to preintervention levels, the CPET group showed significant increases in various indicators postintervention, all of which were higher than those of the conventional group (all *p* < 0.05). This is attributed to increased oxygen intake, enhanced cardiac diastolic function, increased muscle fiber and capillary density, and strengthened muscle strength in major muscle groups, resulting in improved cardiac output, cardiac reserve function, and aerobic capacity in patients. Through comprehensive monitoring of patients' cardiac function, static and dynamic pulmonary function, and exercise capacity throughout CPET, tailored rehabilitation training programs can be developed based on the patient's condition and physical recovery, facilitating rapid improvement in patients' cardiopulmonary function and reducing their fear of exercise after discharge. This contributes to short-term rehabilitation and promotes blood circulation and weight control, thereby reducing high-risk factors such as obesity, which positively impacts long-term prognosis and reduces the incidence of cardiovascular events. Moreover, compared to preintervention levels, the CPET group also showed significant reductions in various cardiovascular risk factors postintervention, such as weight reduction and regulation of lipid metabolism, thus improving the progression of cardiovascular disease.

This study has certain limitations: If hypertension is not controlled, heart failure is likely to occur. Currently, some studies [20–22] have found that combining cardiopulmonary and echocardiographic stress tests as a noninvasive auxiliary method can be used to detect cardiac function in early heart failure patients. This can also improve treatment management, prevent progression to later stages of heart failure, and propose new prevention strategies to help clinicians tailor medical treatment. Currently, “exercise rehabilitation,” as the core of cardiac rehabilitation, is gradually gaining attention from healthcare professionals, and more and more patients are opting for exercise rehabilitation interventions in single-center studies. However, these studies often have small sample sizes and short follow-up times. Further research with larger sample sizes and longer follow-up periods is needed to better understand the efficacy of cardiac rehabilitation for hypertensive patients. Additionally, the rehabilitation mode needs further optimization. The use of mobile networks, sensors, and other hardware and software devices can facilitate data transmission and communication between medical staff and patients, thereby improving the effectiveness of rehabilitation. Moreover, the selected patients in this study were relatively young and had Grade 1 hypertension. Future research should aim to conduct more precise studies targeting different age groups and disease stages. It is hoped that through larger-scale studies and follow-up observations in the future, personalized models of exercise rehabilitation for hypertensive patients under the guidance of cardiovascular physicians can be further explored, making hypertension interventions more comprehensive and accurate, thus improving hypertension control rates and enhancing patients' quality of life.

## Figures and Tables

**Table 1:** General data of the two groups.

**Parameter**	**CPET group (** **n** ** = 35)**	**Conventional group (** **n** ** = 35)**	**χ** ^2^/**t**	**p**
Gender (male/female)	22/10	27/8	0.439	0.582
Age (years)	44.84 ± 2.10	46.74 ± 2.23	−0.617	0.539
Duration of illness (months)	67.16 ± 6.40	67.31 ± 6.22	−0.018	0.986
Hemoglobin (g/L)	130.69 ± 2.70	133.29 ± 2.07	−0.826	0.433
Number of medications	2.3 ± 0.92	2.5 ± 0.94	−0.555	0.581

**Table 2:** Comparison of 24-h dynamic blood pressure before and after intervention in the CPET group and conventional group ($\bar{x}\pm s$,mmHg).

**Group**		**Number**	**rSBP**	**rDBP**	**24hSBP**	**24hDBP**	**dSBP**	**dDBP**	**nSBP**	**nDBP**
CPET Group	Before Intervention	32	154.06±7.36	93.31±6.64	114.13±7.49	87.22±6.55	146.84±7.31	89.13±6.48	141.31±8.27	85.09±7.05
	12 Weeks after Intervention	32	145.72±8.36	87.72±6.68	135.91±7.58	79.31±6.25	158.47±7.88	81.75±6.52	133.19±8.43	77.31±6.80

Conventional Group	Before Intervention	35	154.60±5.97	94.83±5.14	144.37±5.99	88.94±4.99	147.43±5.68	90.94±5.18	141.43±6.91	87.09±5.26
	12 Weeks after Intervention	35	150.40±6.19	90.77±4.92	139.54±6.08	84.91±5.22	142.71±5.80	86.94±5.59	137.14±7.05	82.86±5.63

*t*_1_/*p*_1_			18.713/<0.01	17.643/<0.01	19.323/<0.01	20.150/<0.01	19.264/<0.01	27.863/<0.01	9.423/<0.01	9.307/<0.01
*t*_2_/*p*_2_			4.981/<0.01	29.917/<0.01	13.295/<0.01	27.811/<0.01	21.147/<0.01	28.167/<0.01	5.943/<0.01	7.7735/<0.01
*T*_3_/*p*_3_			-0.330/>0.05	-1.050/>0.05	-0.149/>0.05	-1.218/>0.05	-0.367/>0.05	-1.274/>0.05	-0.063/>0.05	-1.319/>0.05
*T*_4_/*p*_4_			-2.619/<0.05	-2.142/<0.05	-2.174/<0.05	-3.996/<0.05	-2.526/<0.05	-3.507/<0.05	-2.090/<0.05	-3.644<0.05

*Note:* t1, P1 = within-group comparison for CPET group before and after intervention; t2, P2 = within-group comparison for conventional group before and after intervention; t3, P3 = between-group comparison before intervention; t4, P4 = between-group comparison after intervention.

Abbreviations: 24hDBP = 24-h diastolic blood pressure; 24hSBP = 24-h systolic blood pressure; dDBP = nighttime diastolic blood pressure; dSBP = nighttime systolic blood pressure; nDBP = nighttime diastolic blood pressure; nSBP = nighttime systolic blood pressure; rDBP = daytime diastolic blood pressure; rSBP = daytime systolic blood pressure.

**Table 3:** Echocardiographic parameters before and after intervention in CPET group and conventional group ($\bar{x}\pm s$).

**Group**		**Number**	**LVEF(%)**	**LVST(mm)**	**LVPWT(mm)**	**LVEDD(mm)**	**LVMI(g/m)**	**E/A**	**LAD(mm)**
CPET Group	Before Intervention	32	55.78±3.07	16.45±55.5	9.76±1.22	46.10±3.34	82.96±18.93	1.07±0.98	37.37±3.33
	12 Weeks after Intervention	32	57.87±3.32	11.71±16.93	9.62±1.23	45.43±3.51	80.92±19.14	1.12±0.92	36.46±2.61

Conventional Group	Before Intervention	35	56.00±2.90	22.31±76.29	9.61±1.30	46.29±3.11	80.70±17.40	1.06±0.10	37.31±2.47
	12 Weeks after Intervention	35	56.80±3.00	13.65±23.22	9.83±1.29	46.36±3.25	83.55±19.17	1.10±0.09	37.26±2.51

*t*_1_/*p*_1_			-4.067/<0.05	1.012/>0.05	1.507/>0.05	5.507/<0.05	2.082/<0.05	-2.850/<0.05	6.197/<0.05
*t*_2_/*p*_2_			-1.154/>0.05	0.978/>0.05	-1.957/>0.05	-0.709/>0.05	-2.609/<0.05	-1.531/>0.05	0.421/>0.05
*t*_3_/*p*_3_			-0.621/>0.05	0.889/>0.05	1.059/>0.05	-0.489/>0.05	1.028/>0.05	1.028/>0.05	0.215/>0.05
*t*_4_/*p*_4_			2.901/<0.05	-0.842/>0.05	-1.477/>0.05	-2.350/>0.05	-1.182/>0.05	2.287/<0.05	-2.733/<0.05

*Note:* t1, P1 = within-group comparison for CPET group before and after intervention; t2, P2 = within-group comparison for conventional group before and after intervention; t3, P3 = between-group comparison before intervention; t4, P4 = between-group comparison after intervention.

Abbreviations: E/A, ratio of early maximal ventricular filling velocity to atrial maximal ventricular filling velocity; LAD, left atrial diameter; LVEF, left ventricular ejection fraction; LVEDD, left ventricular end-diastolic dimension; LVST, left ventricular interventricular septum thickness; LVMI, left ventricular mass index; LVPWT, left ventricular posterior wall thickness.

**(a):** 

**Group**		**Number**	**FEV1/FVC(%)**	**HR peak (beat per minute)**	**SBP peak(mmHg)**	**DBP peak(mmHg)**	**AT-VO**_**2**_ **(ml/min)**	**Max-vo**_**2**_ **(ml/min)**	**Max-vo**_**2**_ **/kg(ml/(min**∗**kg)**	**AT-VO**_**2**_ **/kg(ml/(min**∗**kg))**
CPET Group	Before Intervention	32	92.50±6.83	117.12±10.75	170.90±10.27	95.45±10.84	1421.54±288.02	1703.44±370.28	21.14±1.85	17.66±0.97
	12 Weeks after Intervention	32	104.00±7.34	124.75±13.33	169.03±13.13	95.34±12.62	1502.29±1.2721	1704.10±351.60	21.67±1.86	19.16±1.70

Conventional Group	Before Intervention	35	92.57±6.74	117.43±12.26	172.00±10.10	96.37±10.61	1462.24±302.04	1760.53±376.78	21.43±1.74	17.79±0.87
	12 Weeks after Intervention	35	94.77±6.549	118.23±13.42	172.86±13.89	99.23±13.56	1469.60±293.82	1737.95±353.30	21.23±1.73	17.93±1.04

*t*_1_/*p*_1_			-14.120/<0.01	-7.480/<0.05	0.958/>0.05	0.056/>0.05	-6.835/<0.05	-0.910/>0.05	-4.707/<0.05	-7.851/<0.05
*t*_2_/*p*_2_			-2.982/<0.01	-0.966/>0.05	-0.291/>0.05	-1.003/>0.05	-0.724/>0.05	2.231/<0.05	1.919/>0.05	-1.254/>0.05
*t*_3_/*p*_3_			-0.043/>0.05	-0.245/>0.05	-0.920/>0.05	-0.727/>0.05	-1.214/>0.05	-1.327/>0.05	-1.324/>0.05	-1.118/>0.05
*t*_4_/*p*_4_			5.436/<0.01	4.845/<0.05	-2.602/<0.05	-2.765/<0.05	0.958/>0.05	-0.822/>0.05	2.079/<0.05	9.398/<0.05

*Note:* t1, P1 = within-group comparison for CPET group before and after intervention; t2, P2 = within-group comparison for conventional group before and after intervention; t3, P3 = between-group comparison before intervention; t4, P4 = between-group comparison after intervention.

Abbreviations: AT-vo2, anaerobic threshold oxygen uptake; AT-vo2/kg, anaerobic threshold kilogram oxygen uptake; DBP peak, peak diastolic blood pressure; FEV1/FVC, forced expiratory volume in 1 s to forced vital capacity ratio; HR peak, peak heart rate; Max-vo2, maximum oxygen uptake; Max-vo2/kg, maximum kilogram oxygen uptake; SBP peak: peak systolic blood pressure.

**(b):** 

**Group**		**Number**	**Max-METS(Mets)**	**AT-Mets(Mets)**	**Mets peak(Mets)**	**AT-VO**_**2**_ **/HR(ml/beat)**	**Max-VO**_**2**_ **/HR(ml/beat)**	**VO**_**2**_ **peak(ml/(min**∗**kg)**	**Power peak(W)**
CPET Group	Before Intervention	32	4.87±0.35	5.05±0.28	6.04±0.53	13.40±3.15	14.66±3.57	17.24±1.27	74.55±8.83
	12 Weeks after Intervention	32	6.14±0.45	5.47±0.49	6.19±0.53	13.25±3.02	13.84±3.43	19.78±2.23	87.12±14.53

Conventional Group	Before Intervention	35	4.98±0.37	5.08±0.25	6.12±0.50	13.81±3.57	15.17±3.90	17.42±1.29	76.49±8.81
	12 Weeks after Intervention	35	5.21±0.42	5.12±0.30	6.07±0.50	13.79±3.43	14.89±3.73	18.22±1.47	78.31±11.54

*t*_1_/*p*_1_			-32.200/<0.05	-7.850/<0.05	-7.850/<0.05	1.707/>0.05	7.020/<0.05	-10.462/<0.05	-8.120/<0.05
*t*_2_/*p*_2_			-0.6024/<0.05	-1.257/>0.05	1.958/>0.05	0.171/>0.05	1.979/>0.05	-5.994/<0.05	-1.691/>0.05
*t*_3_/*p*_3_			-1.184/>0.05	-1.125/>0.05	-1.337/>0.05	-1.105/>0.05	-1.208/>0.05	-1.195/>0.05	-1.912/>0.05
*t*_4_/*p*_4_			8.790/<0.05	9.390/<0.05	2.076/<0.05	-1.528/>0.05	-2.768/<0.05	8.787/<0.05	6.688/<0.05

*Note:* t1, P1 = within-group comparison for CPET group before and after intervention; t2, P2 = within-group comparison for conventional group before and after intervention; t3, P3 = between-group comparison before intervention; t4, P4 = between-group comparison after intervention.

Abbreviations: AT-Mets, anaerobic threshold metabolic equivalent; AT-vo2/HR, anaerobic threshold oxygen pulse; Max-Mets, maximum metabolic equivalent; Max-vo2/HR, maximum oxygen pulse; Mets peak, peak metabolic equivalent; Power peak, peak power; VO2 peak, peak oxygen consumption.

**Table 5:** Comparison of lipid metabolism and body mass index before and after intervention in CPET group and conventional group($\bar{x}\pm s$).

**Group**		**Number**	**LDL-C(mmol/L)**	**TC(mmol/L)**	**TG(mmol/L)**	**BMI(kg/m2)**
CPET Group	Before Intervention	32	3.49±0.46	5.67±0.55	2.37±0.42	28.39±4.59
	12 Weeks after Intervention	32	2.90±0.64	5.13±5.54	1.27±0.38	27.12±4.21

Conventional Group	Before Intervention	35	3.55±0.50	5.64±0.62	2.33±0.41	29.28±4.19
	12 Weeks after Intervention	35	3.36±0.31	5.44±0.44	1.89±0.25	29.20±3.96

*t*_1_/*p*_1_			6.189/<0.01	5.458/<0.01	10.393/<0.01	1.259/<0.01
*t*_2_/*p*_2_			1.911/>0.05	1.490/>0.05	5.110/<0.01	0.899/>0.05
*t*_3_/*p*_3_			-1.066/>0.05	0.527/>0.05	-0.358/>0.05	-0.826/>0.05
*t*_4_/*p*_4_			-9.640/<0.01	0.613/<0.01	7.764/<0.01	-2.082/<0.05

*Note:* t1, P1 = within-group comparison for CPET group before and after intervention; t2, P2 = within-group comparison for conventional group before and after intervention; t3, P3 = between-group comparison before intervention; t4, P4 = between-group comparison after intervention.

Abbreviations: BMI, body mass index; LDL-C, low-density lipoprotein cholesterol; TC, total cholesterol; TG, triglycerides.

## Data Availability

The data that support the findings of this study are available from the corresponding author upon reasonable request.
